# In vitro oogenesis from murine premeiotic germ cells using a new three-dimensional culture system

**DOI:** 10.1038/s41420-023-01577-w

**Published:** 2023-07-31

**Authors:** Lu Wang, Zi-Hui Yan, Tao-Ran He, Hai-Xia Liu, Yu-Kang Li, Yi-Lin Niu, Jun-Jie Wang, Massimo De Felici, Wei Ge, Wei Shen

**Affiliations:** 1 College of Life Sciences, Key Laboratory of Animal Reproduction and Biotechnology in Universities of Shandong, Qingdao Agricultural University, Qingdao, 266109 China; 2 Department of Biomedicine and Prevention, University of Rome Tor Vergata, Rome, 00133 Italy

**Keywords:** Differentiation, Regenerative medicine

## Abstract

A faithful reconstitution of the complete process of oogenesis in vitro is helpful for understanding the molecular mechanisms, genetics, and epigenetic changes related to gametogenesis; it can also be useful for clinical drug screening, disease research, and regenerative medicine. To this end, given the consensus that murine female germ cells initiate meiosis at E13.5, substantial works have reported the successful generation of fertile oocytes using E12.5 female gonads as starting materials. Nevertheless, our data demonstrated that murine germ cells at E12.5 have heterogeneously initiated a meiotic transcriptional program based on a measurement of pre‐mRNAs (unspliced) and mature mRNAs (spliced) at a single-cell level. Therefore, to establish a platform that faithfully recapitulates the entire process in vitro (from premeiotic murine germ cells to fully developed oocytes), we here report a novel three-dimensional organoid culture (3-DOC) system, which successfully induced fully developed oocytes from E11.5 premeiotic female germ cells (oogonia). Compared with 2D culture and other 3D culture methods, this new culture system is more cost-effective and can create high-quality oocytes similar to in vivo oocytes. In summary, our new culture platform provides an experimental model for future research in regenerative medicine and reproductive biology.

## Introduction

In vitro culture systems are important research tools for investigating tissue development and clinical diseases. However, traditional two-dimensional (2D) culture methods may hamper cell communication and cannot display the spatial distribution of tissue structures. Over the past few decades, there has been tremendous development in the research of three-dimensional (3D) culture [1–3]. Compared with 2D culture, 3D culture can contribute to the formation of an organotypic structure called organoid, which can be used to demonstrate the structure and function of organs. The construction of 3D culture models has become an important method of reconstructing disease models in vitro, bringing great hope for regenerative medicine, drug research, and precision medicine [2–4].

Mammalian oogenesis is a complex and delicate process. To obtain functional eggs, female germ cells must undergo a series of crucial events, including primordial germ cell (PGC) specification [5, 6], PGC migration and proliferation [7, 8], meiosis initiation and arrest [9, 10], primordial follicle (PF) formation [11, 12], follicle activation and growth [13], and oocyte maturation and ovulation [14, 15]. The complexity of oogenesis increases the difficulty of in vitro culture. Over the past decade, great progress has been made in the establishment of in vitro culture models of oogenesis. Since 2000, multiple studies have created mature oocytes following heterotopic transplantation of newborn mouse ovaries and in vitro maturation [16–18]. The discovery of induced pluripotent stem cells (iPSCs) in 2006 provided a new source of germ cells [19], and then in 2011 and 2012, primordial germ cell-like cells (PGCLCs) with the capacity for normal spermatogenesis and oogenesis were obtained from iPSCs, respectively, which provided a paradigm for the first step of complete in vitro gametogenesis [20, 21]. In the early protocols of in vitro culture, transplantation was necessary for oogenesis, and it was not until 2016 that complete in vitro reconstruction of oogenesis and spermatogenesis was achieved [22–24]. However, all the above methods used germ cells at or after E12.5 as the materials for in vitro culture. Furthermore, it was reported that some meiotic genes started to be expressed at E12.5 [25], which indicated that the model established from E12.5 gonadal ridges (GRs) might not be as effective as a model from earlier GRs for studying the whole process of oogenesis.

Mammalian embryonic ovaries contain a large number of PGCs, but only a small fraction of them develop into mature oocytes due to apoptosis and follicular atresia [26]. The regulatory mechanisms of oogenesis are currently not well-known, and reconstruction of oogenesis in vitro may help to elucidate these mechanisms. In this study, we focused on germ cells from E11.5 female fetal mice, and established a three-dimensional organoid culture (3-DOC) system that enabled the successful generation of fully developed oocytes under complete in vitro conditions. Through mimicking the in vivo environment and reconstructing the process of oogenesis in vitro, we established a platform to further reveal the mechanism of molecular regulation in early meiosis, oogenesis, and follicle formation, and provided an experimental model for research in reproductive biology and reproductive medicine.

## Results

### Meiosis was initiated in female germ cells before the expression of the meiotic gatekeeper *Stra8*

**Fig. 1:**
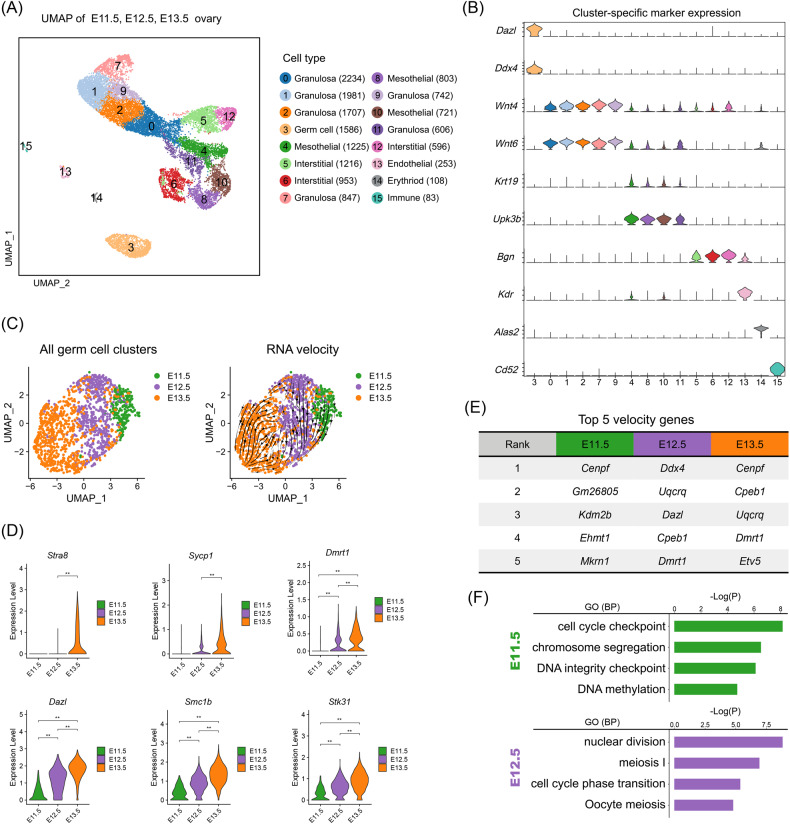
scRNA-seq analysis between E11.5 and E12.5 germ cells. **A** UMAP plot and the cell type identification of GR cells. **B** Marker gene expression profiles in different clusters. **C** RNA velocity streamlines embedded in the UMAP plot colored by time points. **D** Expression levels of genes related to meiosis initiation in E11.5–E13.5 germ cells. **E** The top 5 genes of E11.5–E13.5 germ cells based on their likelihood in the dynamic model of transcription. **F** GO enrichment analysis of marker genes in E11.5 and E12.5 germ cells.

To unveil the transcriptome difference between germ cells at different stages, we firstly performed an integrated analysis of our recently published single-cell RNA seq datasets (E11.5, E12.5, and E13.5) using uniform manifold approximation and projection (UMAP) [27, 28]. After stringent quality control standards to remove low-quality cells and potential doublets (Fig. S1A, see materials and methods for details), we identified 15 cell clusters across three developmental stages (Figs. 1A and S1B), and seven cell types were characterized according to their expression of canonical cell markers (Fig. 1B), including germ cells expressing *Dazl* and *Ddx4* [29], granulosa cells expressing *Wnt4* and *Wnt6* [30], mesothelial cells expressing *Upk3b* and *Krt19* [30], interstitial cells expressing *Bgn* [31], endothelial cells expressing *Kdr* [32], erythroid cells expressing *Alas2* [33], and immune cells expressing *Cd52* [34].

To conduct a focused analysis of germ cells, we extracted the scRNA-seq data of germ cells after the identification of gonadal cells. After UMAP projection, we observed the highest transcriptional heterogeneity in germ cells at E13.5, while E11.5 germ cells showed the lowest transcriptional heterogeneity. Projection of RNA velocity vectors confirmed the developmental trajectories from the top right to the bottom left (Fig. 1C), which was also consistent with the developmental stages (from E11.5 to E13.5). Noteworthy, E11.5 germ cells and E12.5 germ cells were located differently in the UMAP plot, thus suggesting that these germ cells were heterogeneous at the transcription level. To evaluate the meiotic potential of these germ cells at different time points, we then explored the expression of canonical meiotic initiation-related gene expression across the three stages. The results showed that a small number of cells began to express *Stra8* and *Sycp1* as early as E12.5, while other meiotic regulatory genes such as *Dazl*, *Smc1b*, *Stk31,* and *Dmrt1*, showed significantly higher expression in E12.5 germ cells when compared with E11.5 germ cells (Fig. 1D).

To clarify that germ cells at E12.5 had initiated meiotic programs, we further performed RNA velocity analysis to infer cellular fate by leveraging splicing kinetics [35, 36]. By comparing the stage-specific top velocity genes, we found that mitosis-related velocity driving genes were mainly enriched in E11.5 germ cells, while at E12.5, meiotic-related genes were identified in velocity driving genes, such as *Dazl* and *Cpeb1* (Fig. 1E, and Table S6) [37, 38]. Furthermore, we performed GO enrichment analysis on germ cells from different time points. It was found that “meiosis I” and “oocyte meiosis” could be enriched at E12.5 (Fig. 1F), while the E11.5 top enriched RNA velocity genes were related to the GO terms of “cell cycle checkpoint” and “chromosome segregation”, thus representing a mitotic state. In summary, germ cells at E12.5 had initiated a meiotic program even though the meiotic gatekeeper *Stra8* was barely expressed.

### Female germ cells from E12.5 GRs show higher meiotic potential compared with E11.5 germ cells under in vitro conditions

**Fig. 2:**
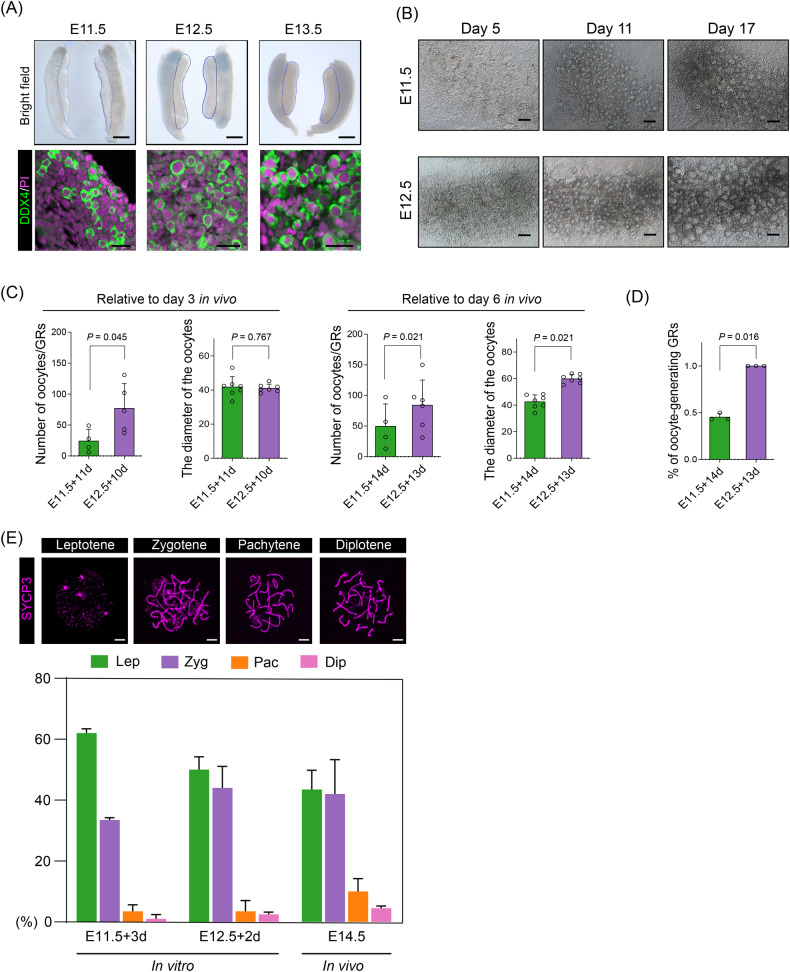
In vitro 2D culture of 11.5 and 12.5 GRs. **A** E11.5–E13.5 germ cell morphology (scale bars = 200 μm) and staining of tissue sections (scale bars = 10 μm). **B** Morphology of oocytes in E11.5 GRs cultured for 5, 11 and 17 days, and oocytes in E12.5 GRs cultured to the corresponding time points. Scale bars = 100 μm. **C** The number and diameter of oocytes in E11.5 and E12.5 GRs cultured for 11 and 10 days (left) and 14 and 13 days (right). **D** Percentages of oocyte-generating GRs after 2D culture. **E** Chromatin spread analysis and statistical analysis of oocytes in different stages of prophase I. Oocytes were labeled by SYCP3 (purple). Scale bars = 10 μm.

Based on our previously established adherent culture methods [39, 40], we next explored the developmental potential of E11.5 and E12.5 female germ cells (Fig. S2A). We firstly collected E11.5 female GRs using *Sry* genotyping because GRs are sexually indistinguishable at this development time point. Morphologically, E11.5 GRs showed an obvious elongated length when compared with E12.5 and E13.5 GRs, and were characterized by a lower number of germ cells (Fig. 2A). We next cultured these GRs under adherent conditions, and representative follicle structures were observed at around Day 11 of in vitro culture (Fig. 2B). However, E11.5 GRs generated lower numbers of follicles when compared with E12.5 GRs after in vitro culture (11 d and 14 d for E11.5 GRs, 10 d and 13 d for E12.5 GRs). Besides, germ cells from E11.5 formed oocytes with significantly smaller diameters after 14 days of in vitro culture (Fig. 2C). We further compared the ratio of oocyte-generating GRs, and the results also confirmed that E12.5 showed a significantly greater ability to produce follicle structures (Figs. 2D and S2B).

As the onset and arrest of meiosis is a crucial event during oogenesis, we then focused on early meiosis and performed chromatin spread analysis on both in vitro and in vivo oocytes at the same stage. The protein synaptonemal complex protein 3 (SYCP3), one component of the synaptonemal complex, was stained for oocyte stage classification [41, 42]. Then oocytes were divided into leptotene, zygotene, pachytene, and diplotene according to the morphology of the synaptonemal complex, and the percentages of oocytes at the four stages were calculated respectively (Fig. 2E). As Fig. 2E indicates, oocytes could enter meiosis, undergo normal homologous synapsis, and develop to the pachytene and diplotene stages after in vitro 2D culture. Nevertheless, most of the oocytes from E11.5 GRs were still in the leptotene stage, which indicated a later meiotic process than that in E12.5 GRs. Taken together, these data verified our scRNA-seq analysis and demonstrated that the onset of meiosis had taken place in some female germ cells at E12.5, which indicated that E11.5 female germ cells were superior for an in vitro culture model considering that they remained mitotic.

### The establishment of 3-DOC enables the proper progression of meiosis for E11.5 female germ cells

**Fig. 3:**
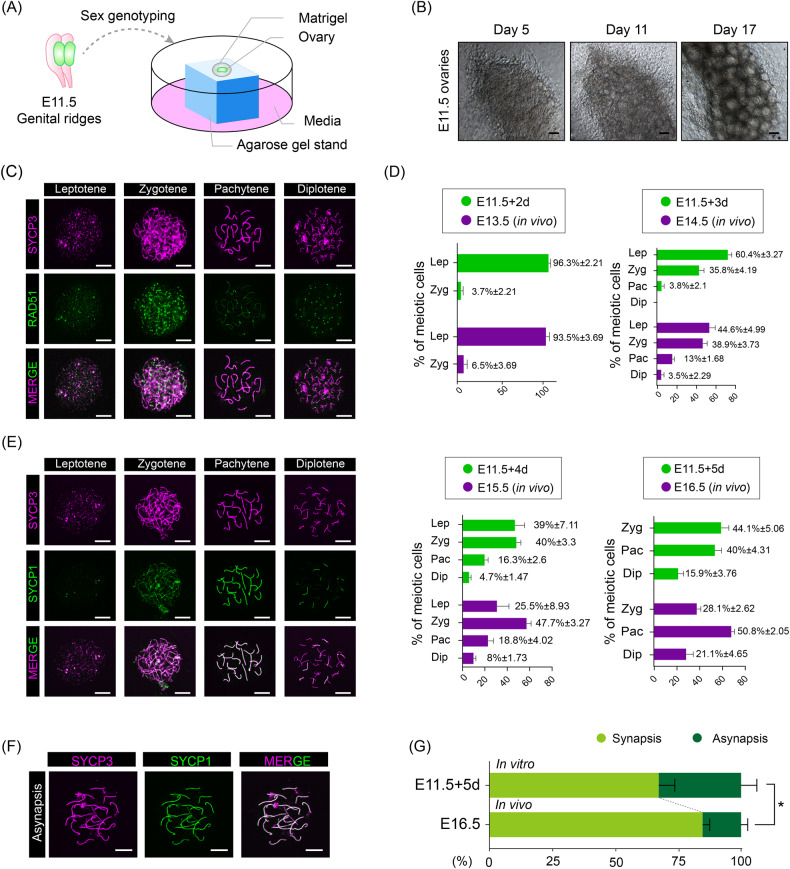
The meiotic process after 3-DOC. **A** Diagram of the 3D culture model. **B** E11.5 fetal mouse GRs cultured for 5, 11, 17 days. Scale bars = 100 μm. **C** Chromatin spread analysis of oocytes in different prophase I stages. Oocytes were labeled by SYCP3 (purple) and RAD51 (green). Scale bars = 10 μm. **D** Statistical analysis of prophase I stages of oocytes in vivo and in vitro. **E** Chromatin spread analysis of oocytes in different stages of prophase I. Oocytes were labeled by SYCP3 (purple) and SYCP1 (green). Scale bars = 10 μm. **F** Chromatin spread analysis of the pachytene oocytes with asynapsis. Scale bars = 10 μm. **G** Percentages of synapsis and asynapsis oocytes in E11.5 + 5 d (in vitro) and E16.5 (in vivo*)* GRs.

To generate fully developed oocytes from premeiotic female germ cells under complete in vitro conditions, we created a new 3D culture method (Fig. 3A). The female GRs of E11.5 in our 3D culture method were cultured on agarose blocks and Matrigel. Noteworthy, our 3-DOC here did not require the application of hanging cell inserts (commonly used consumables for 3D culture), which makes it more cost-effective when compared with other 3D culture methods [43, 44]. After an 11-day 3-DOC culture, E11.5 female germ cells efficiently formed ovarian follicle structures, and of particular note, these ovarian follicle structures showed distinct boundaries, with multiple layers of granulosa cells around the central oocyte at Day 17 of in vitro culture (Fig. 3B). The comparison of the ovarian follicle formation efficiency and ovarian follicle diameter demonstrated that E11.5 female GRs cultured under the current 3-DOC conditions were much more effective in promoting ovarian follicle formation (Fig. S3A).

After 4 days of in vitro culture, we next prepared meiotic chromosome spreads from E11.5 female GRs and the spreads were co-immunostained for the synaptonemal complex marker SYCP3 and homologous recombination marker RAD51. Analyzing RAD51 foci at pachytene verified that under 3-DOC conditions, E11.5 female germ cells showed a comparable number of RAD51 foci to their in vivo counterparts, thus indicating that these E11.5 derived germ cells faithfully recapitulated meiotic recombination (Figs. 3C and S3B). We further compared the progression of meiotic prophase I of E11.5 female germ cells after 2, 3, 4, and 5 days of in vitro 3-DOC culture, and the results showed that those germ cells cultured in the 3-DOC conditions gradually entered meiosis prophase I with prolonged culture; of particular notice, the percentage of germ cells at different meiotic stages resembled their in vivo counterparts (Fig. 3D). As most of oocytes have entered meiosis in E16.5 [45], we further evaluated the homologous chromosome synapsis of the in vitro cultured E11.5 female germ cells by co-staining SYCP3 and SYCP1, and the results showed that approximately 30% of germ cells cultured in vitro showed asynapsis, which was higher when compared with E16.5 female germ cells in vivo (Fig. 3E–G). In general, the majority of germ cells (approximately 70%) underwent normal synapsis under in vitro conditions. Together, these results demonstrated that our established 3-DOC efficiently enabled the progression of meiosis and recapitulated key meiotic events under complete in vitro conditions.

### Prolonged culture of E11.5 female germ cells under 3-DOC conditions recapitulated key events during PF assembly

**Fig. 4:**
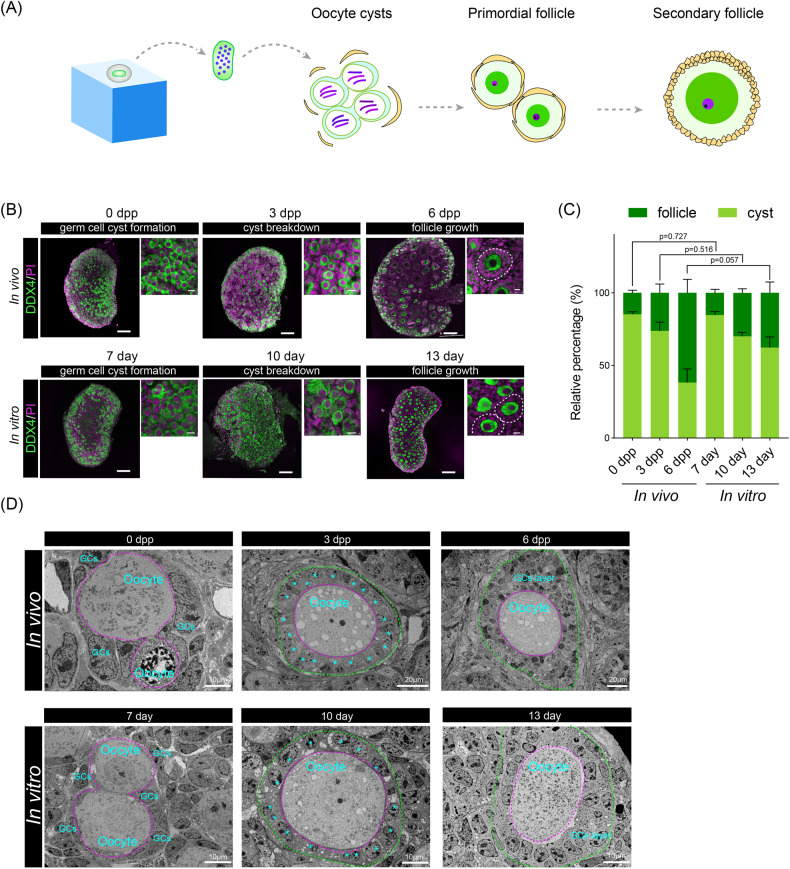
Follicle formation after 3-DOC. **A** Diagram of primordial follicle formation after in vitro 3D culture. **B** Immunofluorescent images of in vitro and in vivo ovaries. Scale bars = 100 μm. **C** The percentages of oocytes in cysts and follicles between in vitro and in vivo ovaries. **D** TEM images of oocytes in cysts and primordial follicles.

During the process of meiosis, germ cell cysts were formed and then underwent programmed cyst breakdown to form PFs (Fig. 4A). To investigate the process of PF assembly of E11.5 female germ cells under 3-DOC conditions, we then performed immunofluorescence on the E11.5 female GRs after being cultured in vitro, and on GRs from postnatal mice at the corresponding time points (Fig. 4B). It was observed that female germ cells under 3-DOC successfully formed germ cell cyst structures after 7 days of in vitro culture. After prolonged culture, these germ cell cyst structures underwent programmed breakdown and finally formed PFs in the center of GRs. To evaluate the progression of PF assembly, we then analyzed the ratio of germ cells within cysts and follicles after 7, 10, and 13 days of 3-DOC culture (Fig. 4C). According to the results of immunofluorescence, a single oocyte surrounded by a single layer of granulosa cells was classified as the oocyte in the follicle, while oocytes in clusters were identified as oocytes in cysts. An accurate estimation method described by Myers, M. et al. was used to count the numbers of oocytes in follicles and cysts [46]. The results showed that female germ cells derived from E11.5 GRs underwent PF assembly resembling their in vivo counterparts (no significance).

To further validate the process of PF assembly in vitro, we performed an ultrastructure analysis of 3-DOC cultured E11.5 GRs (Fig. 4D). The cyst structure can be found in both E11.5 female GRs cultured for 7 days in vitro and ovaries from 0 day post-partum (0 dpp) mice. Meanwhile, the complete structure of follicles that included an oocyte enclosed in a single layer of flattened granulosa cells could be observed in E11.5 female GRs cultured for 10 days in vitro and ovaries from 3 dpp mice, indicating these germ cells formed representative PF structures at this stage. Moreover, in E11.5 female GRs cultured for 13 days in vitro and ovaries from 5 dpp mice, we discovered that some oocytes were surrounded by multiple layers of granulosa cells, indicating they were in the secondary follicle stage (Fig. 4D). Taken together, the results of immunofluorescence and Transmission electron microscopy (TEM) demonstrated that, after 3D culture, the E11.5 female GRs were capable of cyst breakdown and PF assembly.

### Full development of 3-DOC derived premeiotic female germ cells into MII stage oocytes under in vitro conditions

**Fig. 5:**
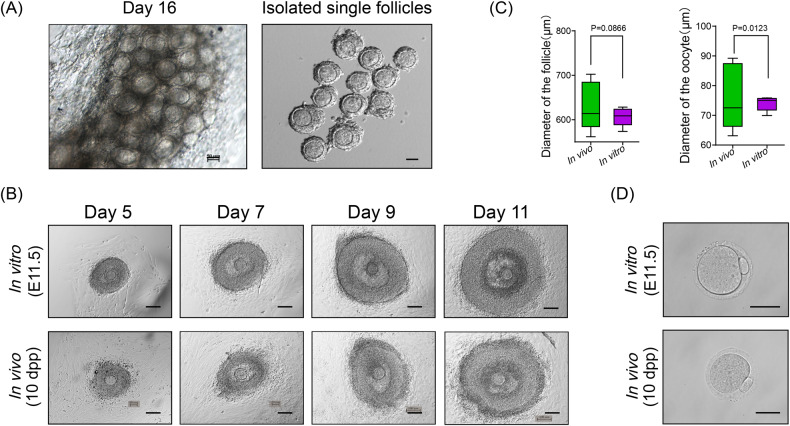
In vitro culture and maturation of oocytes. **A** Images of the in vitro cultured ovaries on Day 16 (left, scale bar = 50 μm) and the isolated single follicles from the in vitro ovaries (right, scale bar = 100 μm). **B** Images of in vitro and in vivo follicles on Day 5–11 of culture. In vitro and in vivo follicles were isolated from the 3D culture ovaries and fetal mouse ovaries respectively, and cultured using a single follicle culture method. Scale bars = 100 μm. **C** The diameters of in vivo and in vitro follicles (left) and oocytes (right) after single follicle culture. **D** Images of mature oocytes after IVM. Scale bars = 50 μm.

After 16 days of 3-DOC culture, E11.5 derived premeiotic female germ cells successfully generated primordial and secondary follicles; we next investigated whether these oocytes could develop beyond the growth stage. To this end, we adopted the two-step culture system established in 1996 by Eppig [47], and isolated the primordial follicles and secondary follicles from ovary organoids after 16–18 days of 3-DOC culture. The follicles had a single layer or multiple layers of granulosa cells, which was similar to their in vivo counterparts (Figs. 5A and S4). We next isolated these single follicles for in vitro single follicle culture using a PVP supplemented medium as described by Morohaku et al., and the results showed that granulosa cells proliferated rapidly during single follicle culture and follicle cavities gradually formed after 7 days of single follicle culture [43] (Fig. 5B). With prolonged culture, the diameter of the follicle cavity increased, and granulosa cells expanded to form a follicular structure. Oocytes featuring well-defined germinal vesicles could also be observed in our in vitro cultured follicles. On day 11 of the in vitro single follicle culture, we observed representative cumulus-oocyte-complexes (COCs), and these oocytes showed comparable diameters to their in vivo counterparts (Fig. 5C). Moreover, to examine whether oocytes were sufficient for maturation after 3D culture, we then cultured oocytes with follicle-stimulating hormone (FSH) and human chorionic gonadotropin (hCG) for 13–17 h [48]. After in vitro maturation (IVM), the oocytes resumed meiosis and the first polar body was extruded, which indicated that oocytes were induced into the MII stage (Fig. 5D). The success of IVM demonstrated the feasibility of our 3-DOC system, which might become a cost-effective alternative method for in vitro oogenesis (Table S7).

## Discussion

Normal oogenesis produces functional oocytes, while abnormal oogenesis can result in multiple diseases related to infertility, such as premature ovarian failure, oocyte maturation defects, and polycystic ovary syndrome [49]. According to the World Health Organization (WHO), it is reported that approximately 48.5 million couples globally are affected by infertility, and assisted reproductive technology is one of the most useful interventions [50, 51]. However, problems remain for women with severe infertility, as the source of high-quality oocytes may be limited and cause ethical issues.

Reconstruction of oogenesis in vitro is an alternative approach to investigating female infertility, and great progress has been made in this area [52]. However, there is still a long way to go before these achievements can be used in clinical reproductive medicine. Nevertheless, any improvement to in vitro experimental models could be of great significance for future clinical applications. To investigate the feasibility of inducing premeiotic female germ cells into mature oocytes in vitro, we selected female germ cells from E11.5 GRs as materials. Oocytes from E11.5 female GRs using the method previously established by our lab [53] showed a delayed process of meiosis compared with those from the E12.5 female GRs and in vivo. Furthermore, the quantity and quality of oocytes from E11.5 female GRs were significantly lower than those from E12.5 GRs at the same stage after culture. These results indicated that the distinct status between E11.5 female GRs and E12.5 female GRs results in different characteristics during in vitro culture.

Recently, with the development of various RNA sequencing technologies, research on embryonic development has entered the "omics era" [54–56]. Using analysis at the single-cell level, the dynamic changes of the entire developmental period can be obtained. The application of new technologies has led to new perspectives and insights into the timing of meiotic initiation [57]. The initiation of meiosis is one of the keys to successful gamete development. It was previously thought that mouse female germ cells start to enter meiosis between E13.5 and E14.5. In our analysis results, some genes related to meiosis initiation such as *Dazl* and *Dpeb1* had already started to be expressed at E12.5, while E11.5 germ cells were still undergoing mitosis. The different expression patterns between E11.5 female germ cells and E12.5 female germ cells might be the reason for the low efficiency of the traditional 2D culture method. Therefore, a new culture system was needed for the culture of E11.5 female GRs.

To improve culture efficiency, we established a novel 3-DOC platform for the in vitro culture of E11.5 female germ cells. As Fig. 3A indicates, the GRs were placed on agarose blocks which were flooded to their upper surface with medium. We also used Matrigel to support cell development during 3-DOC. In recent years, natural extracellular matrices or synthetic hydrogels have been commonly used in cell culture [58, 59]. Matrigel, which is secreted by mouse tumor cells, is a mixture of proteins including collagen IV, laminin, and enactin [60, 61]. Matrigel can mimic the extracellular environment, provide cells with structural support, and promote cell differentiation and development [62]. In our 3-DOC model, we added Matrigel to mimic the in vivo microenvironment and improve cell culture conditions. Unlike oocytes from the 2D culture method, female germ cells from E11.5 GRs showed a similar meiotic process to oocytes in vivo. In addition, oocytes can also undergo PF formation after 3D culture and the structure of follicles was similar to those from in vivo ovaries.

In female mice, the diameter of oocytes during meiotic arrest is 15–20 μm. When the diameter reaches 75 μm or more, the oocytes are considered to be in a state that can be matured in vitro [63]. In our experiments, the oocytes after single follicle culture were greater than 75 μm in diameter. To further verify the quality of our oocytes from the 3-DOC system, we performed IVM after single follicle culture. It was discovered that MII oocytes could be obtained after our 3D culture, and the first polar body could be extruded.

Through the scRNA-seq analysis, we demonstrated the differences between E11.5 and E12.5 germ cells on the meiosis initiation. Then we successfully established a 3-DOC model in this paper and completed the in vitro maturation of oocytes from E11.5 female GRs. Compared with other methods, our method is more cost-effective and can generate high-quality oocytes that closely resemble their in vivo counterparts. Overall, our findings offer a new experimental method for future research in reproductive biology and reproductive medicine.

## Materials and methods

### Animals

The healthy six-week-old CD1 mice used in our experiments were purchased from the Qingdao Daren Fortune Animal Technology Co., Ltd (Qingdao, China). The mice were provided with free access to food and water, and housed under a cycle of 12 h light and 12 h dark. For mating, mice were randomly selected by the random number table method. One male mouse was mated with one or two female mice at 17:00. The presence of a vaginal plug was checked the next morning and the female mice with vaginal plugs were considered as 0.5 day post coitum (dpc) at noon. All the experiments were permitted by the Ethics Committee of Qingdao Agriculture University.

### Isolation of GRs and gender identification

The 11.5 dpc or 12.5 dpc pregnant mice were sacrificed, and embryos were collected from the uteri and washed with normal saline. Female GRs of E11.5 or E12.5 were carefully isolated from the embryos under a stereoscope (Nikon SMZ-1000, Japan) and the mesonephroi were cut off using a 1 mL syringe needle. For E11.5 GRs, the gender cannot be distinguished through morphology. So we performed PCR on the E11.5 embryos and selected the female GRs based on the band of sex-determining gene *Sry*. The primer sequences of *Sry* and the internal reference gene *Gapdh* are listed in Table S1. The collected GRs were washed three times with normal saline for further experiments.

### 2D culture of GRs

The medium was prepared according to Table S2 and balanced at 37°C for 10 min before culture. Subsequently, the collected GRs were washed in medium, transferred to the 24-well plate, and cultured as a whole at 37°C and 5% CO_2_ for three days. Subsequently, half of the medium in the 24-well plate was replaced by the new balanced medium every other day. The entire culture took 17 days in total.

### 3-DOC system of GRs

To construct the 3-DOC system, 2% agarose gel was prepared and sterilized in advance. Before culture, the agarose gel was cut into cubes and placed in the culture medium to replace the water in the gel. We applied Matrigel to our culture system, which was placed on the agarose blocks and balanced in the medium for 30 min. The GRs were then put on the agarose blocks, and the blocks were transferred into the 24-well plate. The medium was the same as that of the 2D culture system, and an appropriate amount of medium was added to ensure that the liquid level reached the upper surface of the agarose blocks.

The GRs were cultured at 37°C and 5% CO_2_ for six days, and the medium was replaced every three days. On the sixth day, we replaced the normal medium with medium containing 5 μM ICI182780 (R&D Systems 1047, USA), which is an inhibitor of estrogen receptors. Half of the medium containing ICI182780 was changed every other day. On the 12th day, we replaced the medium containing ICI182780 with the normal GR culture medium again and the culture was continued until the 17th day.

### Isolation and in vitro culture of follicles

The isolation of follicles was performed when most of follicles reached the stage of secondary follicles with multiple layers of granulosa cells surrounding them. To conduct follicle isolation, the ovary-like tissue was first separated from the surface of agarose blocks and transferred into the isolation medium. The follicle isolation medium was prepared as shown in Table S3. A tungsten needle was then used to isolate follicles carefully. We isolated follicles mechanically and washed them in follicle isolation medium and follicle growth medium in turn. The follicle growth medium was prepared according to Table S4. Before the isolation of follicles, we placed several droplets of follicle growth medium on the dishes, covered them with mineral oil, and equilibrated them for 5 h in the incubator. After follicles were isolated and washed, each follicle was placed in one droplet. The follicles were cultured at 37°C and 5% CO_2_, and half of the medium was changed every two days. Follicle growth took place over a period of 12–14 days.

### Induction of follicle maturation

The maturation medium was prepared as indicated in Table S5. Four droplets of the maturation medium were pipetted on the dishes, covered by mineral oil, and equilibrated in the incubator for 12 h. Follicles with multiple layers of granulosa cells were selected and transferred to the droplets for maturation. Follicle maturation was identified by the extrusion of the first polar body.

### The analysis of scRNA-seq

The scRNA-seq of E11.5–E13.5 gonadal cells used in this paper were previously used by Novogene (Beijing, China) [64] and the data are deposited in the Gene Expression Omnibus (GEO) database under accession number GSE128553. The software CellRanger v6.1.2 was used for quality control of the data and genome alignment according to the CellRanger pipeline.

After obtaining the expression matrix, R v.3.6.3 was used for downstream analysis of scRNA-seq data and doublets were removed by the “DoubletFinder” [65]. The “Seurat” Package v.4.0.0 was used for the aggregation of the data from three groups of GRs based on the official vignette (https://satijalab.org/seurat/index.html) [66]. Uniform Manifold Approximation and Projection was applied for dimension reduction and the function “FindAllMarkers” were used to identify the marker genes of each cluster. We also performed RNA velocity with the “scVelo” [67] package and the RNA velocity streamlines were embedded in the UMAP plot from the “Seurat” package. GO enrichment analysis was performed on Metascape (https://metascape.org/gp/index.html) [68].

### Transmission electron microscopy

To prepare TEM samples, the ovary-like tissue was initially fixed in 2.5% glutaraldehyde and Osmium tetroxide. Samples were subsequently dehydrated in an alcohol and acetone solution, and finally embedded in resin. After resin solidification under different temperatures, the samples were sectioned at approximately 70 nm. Then the slides were treated with uranyl acetate for 15 min and lead citrate for 8 min, in turn. After staining, the slides were observed under an electron microscope (Hitachi HT7700, Japan).

### Immunofluorescence

The GR samples were first collected, fixed in 4% paraformaldehyde at 4°C overnight and then washed with water for 4 h. After dehydration in an alcohol solution, the samples were finally embedded in paraffin and sliced at a thickness of 5 μm. Subsequently, the slides were placed in the 60°C incubator for 2 h and deparaffinized with xylene, alcohol, and PBS solution. For antigen retrieval, slides were treated with 0.01 M sodium citrate solution for 10 min at 96°C. After cooling to room temperature, slides were treated with TBS and TBST for 5 minutes, respectively. Blocking buffer (0.05 M TBS supplemented with 3% bovine serum albumin and 10% goat serum) was added to block the slides. After blocking for 30 min, the slides were incubated with the anti-DDX4 antibody (Abcam ab27591, UK) at 4°C overnight. We then washed the slides three times with TBST. After washing, the slides were incubated with the secondary antibody at 37°C for 45 min. The Alexa Fluor^®^ 488 goat anti-Rabbit antibody (Abcam ab150077, UK) was used as the secondary antibody. Slides were washed with TBST after incubation and 60 μL propidium iodide (PI) was added to each slide for nuclei staining. Finally, slides were washed with PBS and an antifade mounting medium (Boster, AR1109, Wuhan, China) was added before mounting. Photos were taken under a fluorescence microscope (Olympus BX51, Japan) or LSCM (Leica TCS SP5 II, Germany).

### Chromosome spread analysis

To perform chromosome spread analysis, the GRs were isolated, washed with normal saline, and finally placed in a hypo-extraction buffer, which contained 30 mM Tris, 50 mM sucrose, 17 mM citric acid, 5 mM EDTA, 2.5 mM DTT, and 1 mM PMSF. After treatment with the hypo-extraction buffer for 1 h at 4°C, the GRs were placed in a droplet of 0.1 M sucrose on a slide and torn using forceps. Then 500 μL PFA was added to fix the cells at room temperature overnight. The slides were air-dried the next day and washed with 0.04% Photo-Flo for 4 min. Subsequently, the slides were washed with TBS and blocked with blocking buffer (4 mL TBS supplemented with 12 mg bovine serum albumin, 40 μL goat serum, and 0.2 μL Triton-X-100) for 1 h at 37°C. After blocking, the slides were incubated with primary antibodies at 37°C for 8 h. The primary antibodies used in the experiment included anti-SYCP3 (Abcam ab97672, UK), anti-SYCP1 (Abcam 15090, UK), and anti-RAD51 (Abcam ab133534, UK). Then the slides were washed with TBS and incubated with the secondary antibodies at 37°C for 2 h. After incubation and TBS washing, Hoechst 33342 was used to stain nuclei for 8 min. The slides were washed with PBS after nucleus staining and mounted using an antifade mounting medium. Images were taken under a fluorescence microscope (Olympus BX51, Japan) or LSCM (Leica TCS SP5 II, Germany).

### Statistical analysis

The software ImageJ 1.53c was used for image analysis. At least three replicates were performed in each experiment. Two-sided *t* test and *F* test were conducted using Graphpad Prism 5. The data in this paper are presented as mean ± SD. * and ** represent *p* < 0.05 and *p* < 0.01, respectively.

## Supplementary information

Supplemental material

## Data Availability

The scRNA-seq data analyzed during the current study are available in the Gene Expression Omnibus (GEO) database under accession number GSE128553. Other data are included in this published article and supplementary information files.

## Code availability

Main scripts used for scRNA-seq data processing and analyses are available at this link: https://github.com/Flyingcattle/mouse-ovary-organoids.
