# Supplementary material for: In vitro oogenesis from murine premeiotic germ cells using a new three-dimensional culture system
Source: Cell Death Discov. 2023 Jul 31;9:276. doi: 10.1038/s41420-023-01577-w (PMC10387482; doi:10.1038/s41420-023-01577-w)
Supplement: Supplementary file 1 — Supplemental material [file 41420_2023_1577_MOESM1_ESM.docx]

**Supplementary materials**

**Fig. S1**


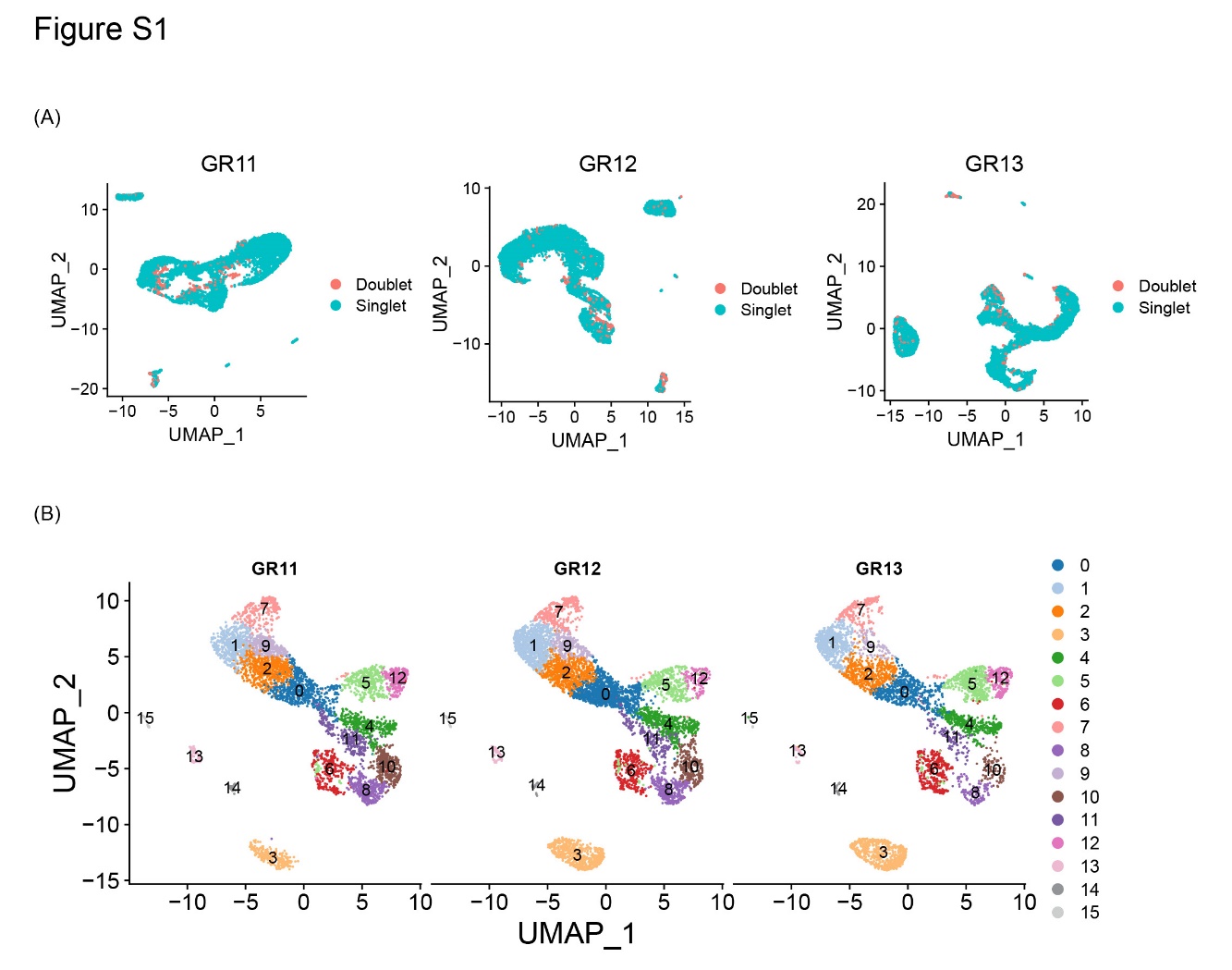


**Fig. S1. Doublet filtering and UMAP analysis of data from E11.5–13.5 GRs.** (A) Doublet filtering of cells from E11.5–13.5 GRs. (B) UMAP plot of cells from E11.5–13.5 GRs.

**Fig. S2**


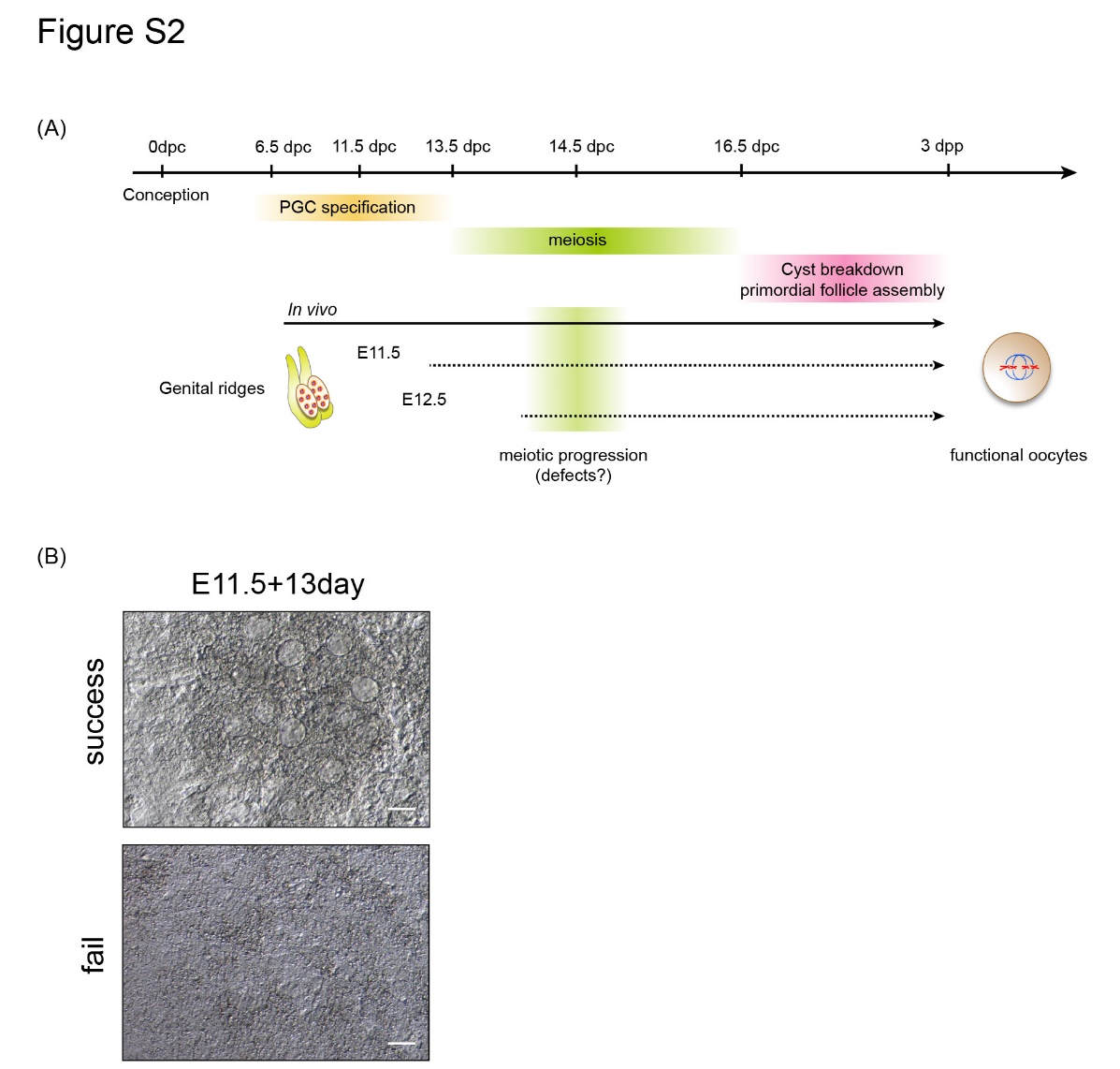


**Fig. S2. Design and results of *in vitro* 2D culture.** (A) Diagram of germ cell development and experimental design. (B) Images of *in vitro* cultured E11.5 GRs that generated or failed to generate oocytes on Day 13. Scale bars = 50 μm.

**Fig. S3**
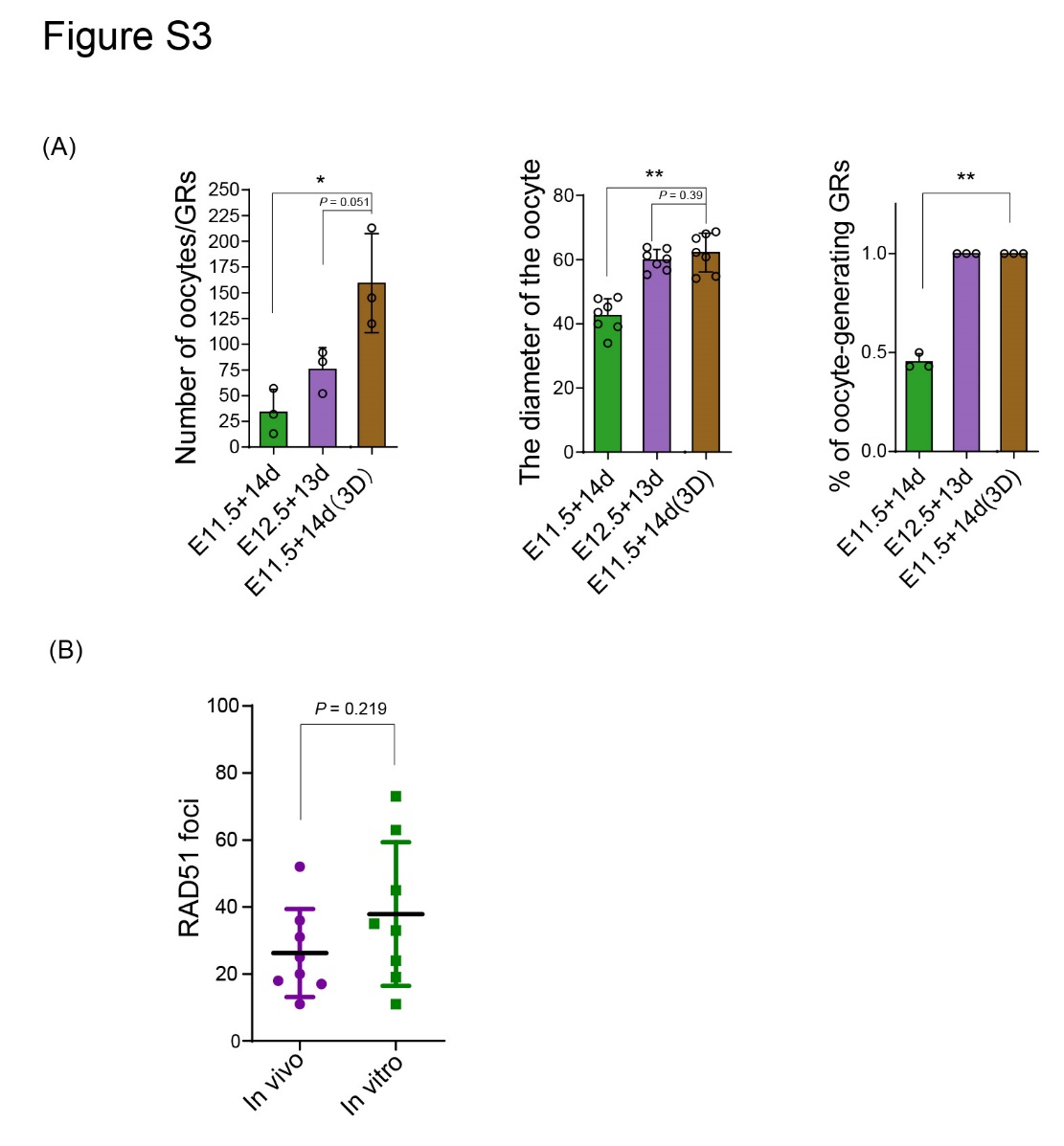


**Fig. S3. Comparison of *in vivo* oocytes, 2D culture oocytes, and 3-DOC oocytes.** (A) Comparison of the number and diameter of oocytes, and percentages of oocyte-generating GRs between 2D culture and 3-DOC. (B) Comparison of the RAD51 loci between *in vivo* oocytes and 3-DOC oocytes during pachytene.

**Fig. S4**


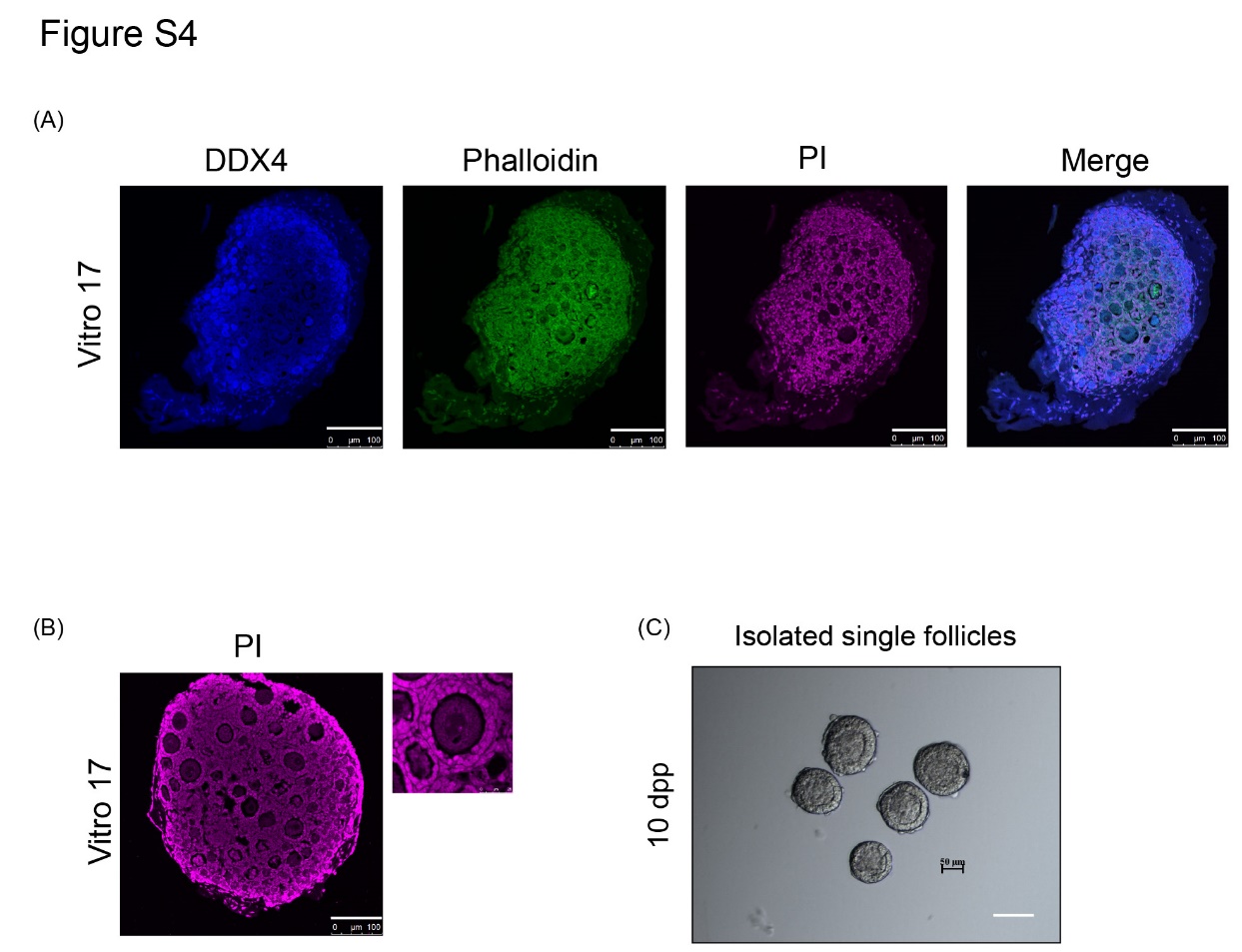


**Fig. S4. 3-DOC follicle structure.** (A) Immunofluorescent images of 3-DOC ovaries on Day 17. Scale bars = 100 μm. (B) Immunofluorescent images of 3-DOC secondary follicles on Day 17. Scale bars = 100 μm. (C) The isolated single follicles from the *in vivo* ovaries on 10 dpp. Scale bars = 100 μm.

**Table S1 The primer sequences of Sry and Gapdh**

| **Genes** | **Sequence (5'-3')** |
| --- | --- |
| *Sry-F’* | TTGTCTAGAGAGCATGGAGGGCCATGTGAA |
| *Sry-R’* | CCACTCCTCTGTGACACTTTAGCCCTCCGA |
| *Gapdh-F’* | AGGTCGGTGTGAACGGATTTG |
| *Gapdh-R’* | TGTAGACCATGTAGTTGAGGTCA |

**Table S2 Medium formulations for GR culture**

| **Reagents** | **Concentration (V/V)** | **Amounts in 10 mL system** |
| --- | --- | --- |
| α-MEM | 94.80% | 9 480 μL |
| FBS | 5% | 500 μL |
| Vitamin C (150 mM) | 0.10% | 10 μL |
| Penicillin-Streptomycin | 0.10% | 10 μL |

**Table S3 Follicle isolation medium formulations**

| **Reagents** | **Concentration (V/V)** | **Amounts in 10 mL system** |
| --- | --- | --- |
| L-15 Medium | 94.90% | 9 490 μL |
| FBS | 5% | 500 μL |
| Penicillin-Streptomycin | 0.10% | 10 μL |

**Table S4 Follicle growth medium formulations**

| **Reagents** | **Concentration (V/V)** | **Amounts in 10 mL system** |
| --- | --- | --- |
| α-MEM | 94.70% | 9 470 μL |
| FBS | 5% | 500 μL |
| PVP | 2% | 0.2 g |
| FSH (0.1 IU/mL) | 0.10% | 10 μL |
| Vitamin C (150 mM) | 0.10% | 10 μL |
| Penicillin-Streptomycin | 0.10% | 10 μL |

**Table S5 Maturation medium formulations**

| **Reagents** | **Concentration** |
| --- | --- |
| α-MEM | 89% |
| FBS | 10% |
| FSH | 0.1 IU/mL |
| HCG | 1.2 IU/mL |
| Penicillin-Streptomycin | 0.10% |

**Table S6 Enriched genes of E11.5-E13.5 germ cells based on their likelihood in the dynamic model of transcription**

| GR11 | GR12 | GR13 |
| --- | --- | --- |
| Cenpf | Ddx4 | Cenpf |
| Gm26805 | Uqcrq | Cpeb1 |
| Kdm2b | Dazl | Uqcrq |
| Ehmt1 | Cpeb1 | Dmrt1 |
| 4932438A13Rik | Dmrt1 | Etv5 |
| Mkrn1 | 4932438A13Rik | Stk31 |
| Pml | Qser1 | Dazl |
| Cpeb1 | Smc1b | Helz |
| Ddx4 | Tsen34 | Ddx4 |
| Sash1 | Prelid3a | Prelid3a |
| Tle4 | Scml2 | Mkrn1 |
| Jak1 | Stk31 | Qtrt1 |
| Tes | Camk2g | Cdc6 |
| Dennd5a | Qtrt1 | Zfp42 |
| Tomm40 | Map7d2 | Tsen34 |
| Aurka | Fam117b | Ssbp4 |
| Cdc26 | Ehmt1 | Fstl1 |
| Cep57 | Kdm2b | Gm1673 |
| St3gal5 | Dennd5a | Map7d2 |
| Wdr26 | Tomm40 | Stra8 |
| Zfp42 | Gm1673 | Pml |
| Ctnnal1 | Eps15 | Aurka |
| Usp10 | Mkrn1 | Cdc26 |
| Klhl13 | Btbd7 | Txnrd1 |
| Spast | Usp3 | Ina |
| Bod1l | Etv5 | Rnf130 |
| Ptpre | Dcaf5 | Ehmt1 |
| Ptpn3 | Add3 | Ctnnal1 |

| Spdl1 | Cenpf | Camk2g |
| --- | --- | --- |
| Elovl6 | Fut8 | Qser1 |
| Socs2 | Aqr | Aqr |
| Pdk1 | Zfp42 | Fbxl17 |
| Ntrk2 | Hmgn5 | Rnf212b |
| Txnrd1 | Adam10 | Usp10 |
| Dtl | Cdkn2a | Dennd5a |
| Ammecr1l | Txnrd1 | Dtl |
| Cenpu | Dtnbp1 | Smc1b |
| Fut9 | Eea1 | Rec8 |
| Agfg2 | Wdr26 | Osbpl6 |
| Flot2 | Smc6 | Socs2 |
| Efcab2 | Ppp1r13b | Kdm2b |
| Ncapg2 | Nectin2 | Brca1 |
| Mt1 | Ssbp4 | Arid1a |
| Gnas | Ptprf | Etv4 |
| Slc25a13 | Ctnnbip1 | Nectin2 |
| Chmp4c | Tfap2c | Arid5b |
| 9330185C12Rik | Arid5b | Jarid2 |
| Srsf12 | Osbpl6 | Xrn2 |
| Arid5b | Cdca2 | Tomm40 |
| Mcm10 | Rbm47 | Satb1 |
| Miip | Spdl1 | Hmg20b |
| Mep1b | Sash1 | Rnf125 |
| Tipin | Aurka | A830018L16Rik |
| Csnk1g3 | Ocrl | Pik3cd |
| Mtf2 | Hmg20b | Usp3 |
| Orc6 | Ammecr1l | Cep57 |
| Cdca2 | Ncapg2 | Pura |
| Aqr | Cdc6 | Add3 |
| Snx7 | Etv4 | Dtnbp1 |
| Hmgn5 | Epn2 | Iqsec1 |
| Ntn1 | Cdc26 | Tle4 |
| Brca1 | Adam22 | Epb41 |
| Gm11747 | Pml | Macrod1 |
| Zcchc14 | Plagl1 | Cdca2 |
| Cd59a | Zswim1 | Ulk2 |
| E2f2 | Tmx4 | Ccm2 |
| Pecam1 | St3gal5 | Nasp |
| Tlcd1 | Denr | Plagl1 |
| Pdia3 | BC048679 | Mcm10 |
| Alg13 | Pik3cd | Caprin1 |
| Ctbp2 | Jak1 | Utp20 |
| Eif4g3 | Stag3 | Btbd7 |
| Cdc6 | Dmc1 | Eif4g3 |
| Dgkz | Cotl1 | Pum2 |
| Plekhg5 | Usp10 | Eps15 |
| Fam81a | Fstl1 | Dpysl2 |
| Folr1 | Phf21a | Prr11 |
| Rab27a | N4bp2 | Fam117b |
| Ddx17 | A830018L16Rik | Pif1 |
| Otud7b | L1td1 | Tnnt1 |
| Slc1a3 | Socs2 | Eif4h |
| Igf2bp2 | Mt1 | Zfp462 |
| Rbm47 | Dgkz | Chd7 |
| Auts2 | Miip | Uaca |
| Ubap2l | Rad51c | Orc6 |
| Nasp | Ina | Slc2a3 |
| Rcc1 | Spast | Dcaf5 |
| Galm | Cdh3 | L1td1 |
| A830018L16Rik | Bod1l | Kdm1b |
| Helz | Cntln | Pomgnt1 |
| Zfp981 | Top2b | Ctbp2 |
| Caprin1 | Ctbp2 | 4932438A13Rik |
| Uaca | Ptpn3 | Wdr73 |
| Fbxl20 | Nme7 | Klhl13 |
| Mpped2 | Pdcd4 | Hmcn1 |
| Tex15 | Pdia3 | Ammecr1l |
| Abhd16a | Eif4h | Ncapg2 |
| Zfp985 | Elovl6 | Mael |
| Msh2 | Rab27a | Mier1 |
| Slc12a2 | Zfp746 | Igf1r |

**Table S7 Developmental ability of oocytes differentiated from PGCs after growth in vitro**

| Materials | Condition for organ culture | No. of cultured gonads | No. of cultured follicles | No. of survived follicles | | No. of collected COCs | No. of oocytes matured into MII |  |
| --- | --- | --- | --- | --- | --- | --- | --- | --- |
| GRs12.5 | FBS/5 μM ICI | 6 | 312 | 223 (71%) | 178 (80%) | | 168 (94%) | Data from Morohakua et al. (2016) |
| GRs11.5 | FBS/5 μM ICI | 9 | 128 | 13(10.2%) | 13(100%) | | 2(15.4%) |  |
